# Traumatic Intraperitoneal Bladder Rupture Presenting With Massive Ascites and Acute Kidney Injury Following a Ground-Level Fall

**DOI:** 10.7759/cureus.87550

**Published:** 2025-07-08

**Authors:** Tala Nasrini, Blaine Traylor, Nathan Chow, Shaun K Yang, Mitch F Chlopek, Wayne A Martini

**Affiliations:** 1 Internal Medicine, Mayo Clinic Arizona, Phoenix, USA; 2 Emergency Medicine, Mayo Clinic Arizona, Phoenix, USA

**Keywords:** abdominal injuries, acute kidney injury, ascites, bladder injuries, diagnostic imaging, intraperitoneal rupture, paracentesis, urinary bladder

## Abstract

A previously healthy 39-year-old man presented with progressive abdominal distension and discomfort one week after a high-velocity ground-level fall. He initially sought care elsewhere but was discharged without a definitive diagnosis. On presentation to our institution, he exhibited signs of acute kidney injury, abdominal compartment syndrome, and elevated intra-abdominal pressure. Point-of-care paracentesis revealed 7.9 L of fluid with markedly elevated creatinine, consistent with urinary ascites. A CT urogram confirmed bladder dome rupture. The patient underwent exploratory laparotomy with successful bladder wall and peritoneal repair. Postoperatively, he improved without the need for dialysis. This case illustrates an uncommon but critical diagnosis of intraperitoneal bladder rupture following blunt abdominal trauma, masquerading as acute kidney injury due to reverse autodialysis. Recognition of urinary ascites and early surgical intervention can prevent misdiagnosis and irreversible renal injury.

## Introduction

Intraperitoneal bladder rupture (BR) is a rare but potentially life-threatening complication of blunt abdominal trauma, accounting for a minority of bladder injuries, which themselves represent less than 2% of all abdominal trauma cases [1]. BR can be classified as intraperitoneal, extraperitoneal, or combined. While most extraperitoneal injuries are associated with pelvic fractures and are often managed conservatively, intraperitoneal injuries typically require surgical repair and are more likely to occur when the bladder is distended at the time of trauma [2,3].

Although frequently overlooked and misdiagnosed due to its nonspecific clinical manifestations, such as abdominal pain, distension, oliguria, and features of acute kidney injury, BR should remain on the differential in the context of blunt trauma. Despite its clinical importance, delayed intraperitoneal BR remains infrequently reported in the literature, highlighting the diagnostic challenge posed by its nonspecific presentation.

Notably, approximately 10% of BR cases are not associated with pelvic fractures, and isolated intraperitoneal BR due to blunt trauma represents less than 2% of cases [1].

A unique pathophysiological consequence of intraperitoneal BR is “reverse autodialysis,” in which urine in the peritoneal cavity is reabsorbed, leading to elevated serum creatinine levels that mimic intrinsic renal failure [4].

This case is particularly notable for being an isolated intraperitoneal BR following blunt abdominal trauma in a motor vehicle accident, without any pelvic fracture. It underscores the importance of clinical suspicion, especially when initial imaging is inconclusive, and highlights the utility of early diagnostic paracentesis with ascitic fluid creatinine analysis in patients with unexplained ascites and renal dysfunction.

## Case presentation

A 39-year-old man with no significant past medical history presented to the emergency department with a one-week history of progressive abdominal swelling and discomfort. His symptoms began acutely following a fall sustained while sprinting around a truck on gravel; he slipped and landed forcefully on his abdomen. He described the sensation as feeling like he had “ruined something,” with ongoing abdominal distension and pain since the injury. He denied vomiting or fevers but reported profuse watery diarrhea and a marked decrease in urinary output over several days. He also experienced mild shortness of breath, which he attributed to abdominal pressure and pain during deep inspiration.

Initially, he sought care at a local hospital where he received intravenous fluids for presumed dehydration and underwent two CT scans; however, no diagnostic paracentesis was performed. Dissatisfied with his care, he discharged himself after three days and presented to our institution for further evaluation.

He denied any history of genitourinary disease, prior abdominal surgery, or liver disease. The only chronic condition reported was an umbilical hernia diagnosed previously, which had never required surgery. Socially, he reported occasional alcohol consumption, typically five to six servings of whiskey on one or two evenings per month. He did not smoke or use recreational drugs. There was no family history of renal, hepatic, or autoimmune diseases. His medication history included only as-needed ibuprofen for musculoskeletal pain related to his physically demanding occupation. He reported allergies to morphine and oxycodone but tolerated fentanyl without issue.

On examination, he appeared uncomfortable and had a markedly distended, tense abdomen with diffuse tenderness to palpation, but no peritoneal signs. There was no palpable mass or hepatosplenomegaly. Vital signs were notable for a blood pressure of 150/95 mmHg, a heart rate of 120 beats per minute, a respiratory rate of 28 breaths per minute, and an oxygen saturation of 90-95% on room air. He was afebrile. There was an irreducible umbilical hernia without signs of strangulation. Initial laboratory studies revealed acute kidney injury as well as other mild derangements (Table 1).

**Table 1:** Patient’s initial emergency department results

Test	Result	Normal range
Blood urea nitrogen	67.6 mmol/L (H)	2.5-7.1 mmol/L
Serum creatinine	7.91 mg/dL (H)	0.6-1.2 mg/dL
Sodium	132 mmol/L (L)	135-145 mmol/L
Chloride	95 mmol/L (L)	98-106 mmol/L
Bicarbonate	17 mmol/L (L)	22-29 mmol/L
Anion gap	20 (H)	8-16
Alanine aminotransferase	71 U/L (H)	7-56 U/L
Alkaline phosphatase	68 U/L	40-129 U/L
Total bilirubin	0.4 mg/dL	0.1-1.2 mg/dL
Hemoglobin	179 g/L (H)	135-175 g/L
Hematocrit	51.9% (H)	38.8-50%
Platelet count	523 × 10⁹/L (H)	150-450 × 10⁹/L
White blood cell count	15.2 × 10⁹/L (H)	4.0-11.0 × 10⁹/L
Lactate	1.2 mmol/L	0.5-2.2 mmol/L
Lipase	48 U/L	<160 U/L
Ammonia	22 µmol/L	15-45 µmol/L

A diagnostic paracentesis was performed with removal of 7.9 L of straw-colored fluid. Fluid analysis revealed an extremely elevated creatinine concentration, consistent with urinary ascites. Albumin was administered intravenously (50 g) post-paracentesis to mitigate intravascular volume shifts. Urinalysis was ordered alongside post-void residual assessment and protein-to-creatinine ratio.

**Figure 1:**
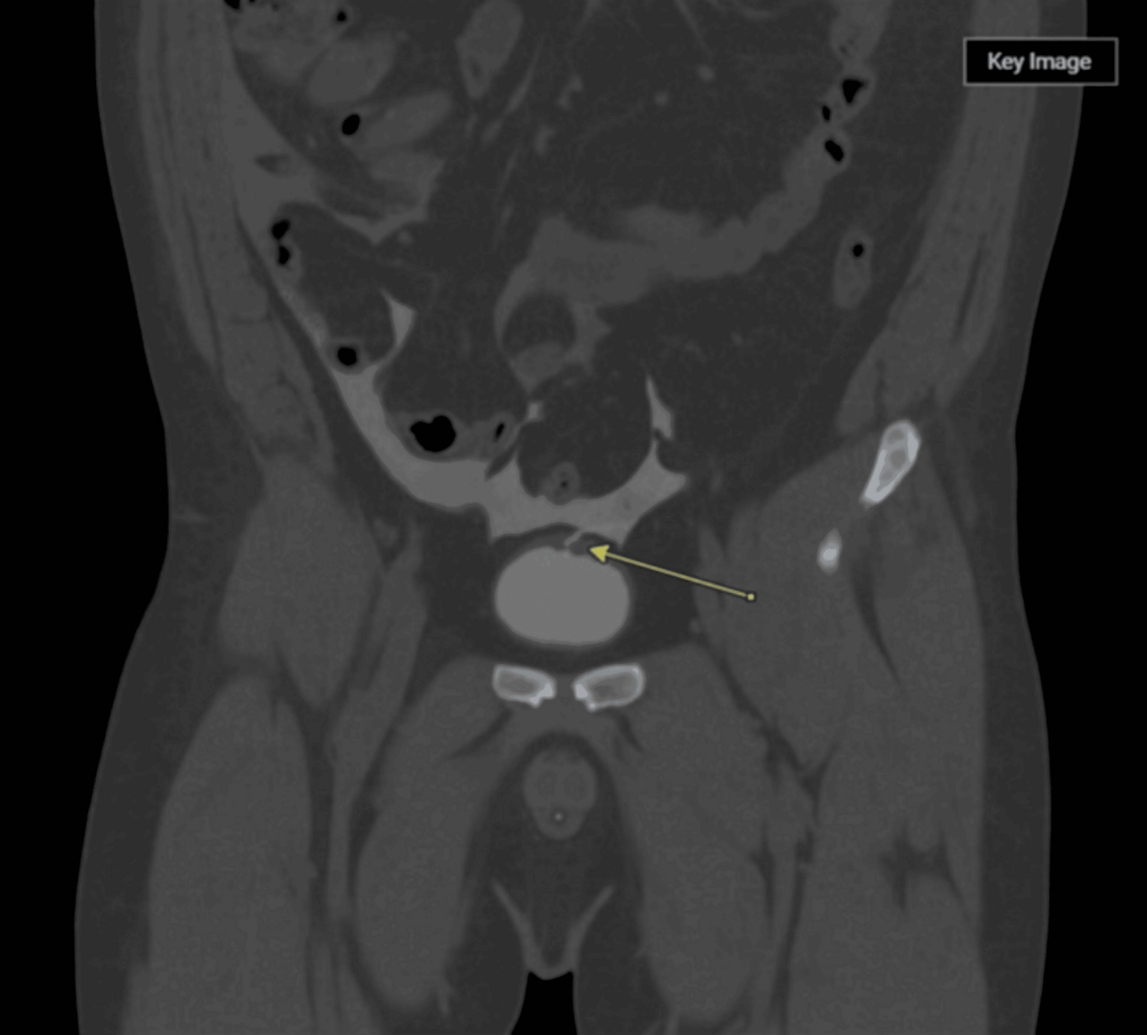
Contrast-enhanced coronal CT scan The yellow arrow is pointing to a defect in the dome of the bladder wall, which appears to be extravasating contrast into the peritoneal cavity, consistent with intraperitoneal BR. BR, bladder rupture

A contrast-enhanced CT urogram demonstrated intraperitoneal fluid surrounding the bladder and a defect at the bladder dome (Figure 1). This radiographic evidence was concordant with suspected intraperitoneal BR. Blood cultures were drawn and remained negative. Viral hepatitis serologies were also ordered to exclude chronic liver disease as a confounding factor for ascites, but were nonreactive. Despite severe azotemia, the nephrology team determined there was no immediate indication for dialysis given the preserved mental status and improving clinical parameters post-paracentesis.

At the time of presentation, the differential diagnosis for this patient’s progressive abdominal distension and acute kidney injury included intraperitoneal BR, bowel perforation with peritonitis, spontaneous bacterial peritonitis, decompensated cirrhosis, intra-abdominal malignancy with ascites, and nephrotic syndrome with third spacing.

Decompensated liver disease was considered due to the presence of ascites; however, the patient had no known history of chronic liver disease, stigmata of cirrhosis, or risk factors for viral hepatitis. Liver function tests were largely unremarkable, and viral hepatitis serologies were negative. There was also no splenomegaly or evidence of portal hypertension on imaging.

Nephrotic syndrome was unlikely in the absence of significant proteinuria or hypoalbuminemia. Bowel perforation was considered given the history of trauma and abdominal pain, but the absence of peritoneal signs, systemic toxicity, or pneumoperitoneum on imaging argued against it. Spontaneous bacterial peritonitis was ruled out by the patient’s immunocompetent status, lack of hepatic disease, and a noninflammatory profile on ascitic fluid analysis.

The marked elevation of creatinine in the ascitic fluid, in combination with urinary retention and diminished urine output, strongly supported urinary ascites secondary to intraperitoneal BR. The presence of reverse autodialysis, where reabsorption of urinary solutes across the peritoneum falsely elevates serum creatinine, further confirmed this diagnosis. The CT urogram demonstrated a focal defect in the bladder dome, clinching the diagnosis of traumatic BR.

Upon confirmation of intraperitoneal BR, the urology team proceeded with urgent operative management. After obtaining informed consent, the patient underwent exploratory laparotomy under general anesthesia. A midline incision was made from the umbilicus to the pubic symphysis. Entry into the peritoneal cavity revealed minimal residual intraperitoneal fluid, as the majority had been evacuated via prior paracentesis. The bladder was mobilized from the abdominal wall and pelvic sidewalls using electrocautery. A large 8 cm tear was identified in the peritoneum overlying the posterior bladder wall and dome. This defect was closed in a running fashion using 3-0 Vicryl sutures.

To inspect the mucosal surface, a cystotomy was performed on the anterior bladder wall. This revealed a 2-3 cm mucosal defect near the bladder dome, which was also closed with 3-0 Vicryl in a running fashion. The anterior bladder incision was then closed in two layers using 2-0 and 3-0 Vicryl for a watertight multilayer closure. The initial intraoperative catheter was exchanged for an 18 French two-way Foley catheter with 10 mL of sterile water instilled in the balloon. The bladder was retrograde-filled with 200 mL of saline to confirm watertight closure. A surgical drain was placed in the left lower quadrant and secured with a nylon suture. The abdominal fascia was closed using a 0-loop PDS suture, followed by layered dermal and skin closure with chromic and Monocryl sutures, respectively. Local anesthesia was provided using 0.25% bupivacaine.

Postoperatively, the patient was managed with nil per os status and nasogastric decompression until bowel function resumed. Empiric ceftriaxone was initiated in the perioperative period and later transitioned to prophylactic nitrofurantoin to be continued until catheter removal. The patient experienced steady improvement in pain, bowel function, and laboratory parameters over the next 72 hours, obviating the need for renal replacement therapy. The patient’s postoperative course was uncomplicated. His nasogastric tube was discontinued on postoperative day two following the return of bowel function, and his diet was successfully advanced to regular intake without gastrointestinal symptoms. He reported progressive improvement in abdominal discomfort and mobility.

Renal function improved steadily without the need for dialysis. Serum creatinine levels decreased from 7.91 µmol/L on admission to 1.52 µmol/L by the time of discharge, consistent with resolution of the reverse autodialysis phenomenon associated with urinary ascites. The Jackson-Pratt drain, placed intraoperatively, was removed prior to discharge after demonstrating minimal output. The Foley catheter was left in place to allow for bladder decompression and optimal healing of the surgical repair.

The patient was discharged home in stable condition on postoperative day three with instructions for oral antibiotic prophylaxis and follow-up in the urology clinic. A voiding cystourethrogram was scheduled for two weeks post-discharge to assess the integrity of the bladder repair before catheter removal. At follow-up, the patient reported full recovery, and imaging confirmed no residual leak. The catheter was successfully removed thereafter.

## Discussion

Intraperitoneal BR is an uncommon but important clinical entity, most frequently caused by high-impact blunt trauma or iatrogenic injury. The bladder dome, due to its mobility and lack of support, is particularly vulnerable to rupture when the bladder is distended at the time of trauma [1,2]. Although BR often accompanies pelvic fractures, isolated intraperitoneal bladder injuries without bony trauma can occur, especially following a direct blow to a full bladder.

Blunt abdominal trauma ranks third after head and chest injuries in trauma incidence and can affect solid organs such as the liver and spleen, as well as hollow organs, including the intestines and bladder [4]. Due to its protected anatomical position within the pelvis, bladder injury is relatively rare, accounting for up to 2% of blunt abdominal traumas [5]. Mechanisms of bladder injury include (i) direct blunt trauma to a distended bladder; (ii) high-energy blunt trauma leading to pelvic fractures with associated bladder injury; and (iii) penetrating or iatrogenic injuries. Motor vehicle accidents, motorcycle collisions, falls from heights, and pedestrian accidents constitute the majority of cases [2,6]. Notably, about 90% of bladder injuries related to blunt trauma occur with pelvic fractures, and extraperitoneal rupture is more common, representing roughly 80% of cases [3,6]. Isolated intraperitoneal BR without pelvic fracture, as in this patient, is rare but clinically significant.

Early diagnosis is critical to avoid complications such as peritonitis, sepsis, and irreversible renal injury. Clinical presentations are often nonspecific, with patients reporting lower abdominal pain, abdominal distension, or signs mimicking an acute abdomen. This can delay recognition, particularly when laboratory findings - such as severe azotaemia - suggest primary renal pathology rather than urinary leakage [3,4]. The phenomenon of “reverse autodialysis,” whereby urinary solutes such as urea and creatinine are absorbed from the peritoneal cavity into the bloodstream, can falsely elevate serum creatinine and urea levels, misleading clinicians [3].

Diagnostic imaging plays a pivotal role in evaluation. Ultrasonography, though accessible, has limited specificity in differentiating free fluid types in trauma cases [5]. CT is the preferred modality in stable patients, allowing identification of both solid organ and hollow viscus injuries. However, non-contrast CT scans may show ascites but often cannot detect bladder wall defects. CT cystography or plain film cystography are considered gold standards for detecting BR but may not be feasible in urgent situations or when contrast is contraindicated [5,7]. In this case, a non-contrast CT urogram revealed intraperitoneal fluid and a focal defect at the bladder dome, supporting the diagnosis.

Paracentesis with creatinine analysis of ascitic fluid was critical here, confirming urinary ascites by demonstrating ascitic fluid creatinine far exceeding serum levels, a hallmark of BR. Therapeutic drainage of nearly 8 L of fluid significantly improved the patient’s symptoms and hemodynamics. Prompt surgical repair remains the definitive treatment for intraperitoneal BR. Direct visualization via cystotomy allows for meticulous closure of serosal and mucosal defects. Postoperative bladder decompression with a Foley catheter is essential for healing, with follow-up cystography to confirm repair integrity before catheter removal [5].

This case highlights several important clinical points: consider BR in patients presenting with abdominal distension and acute kidney injury after trauma, even with low-energy mechanisms and no pelvic fractures; ascitic fluid analysis for creatinine is invaluable in differentiating urinary ascites from other causes; and timely imaging and urologic consultation are crucial for diagnosis and management to prevent severe complications [7,8].

Delayed diagnosis can lead to progressive inflammation, tissue damage, and worsened outcomes, emphasizing the urgency of early intervention [7,8]. Emergency surgery remains a cornerstone worldwide for intraperitoneal BR, even in the current era of advanced imaging and minimally invasive therapies [9,10].

## Conclusions

This case stresses the importance of considering intraperitoneal BR in patients presenting with abdominal distension and acute kidney injury following blunt trauma, even when the mechanism appears low energy and there is no associated pelvic fracture. The presence of urinary ascites and the phenomenon of reverse autodialysis can obscure the diagnosis by mimicking intrinsic renal failure. Early recognition through paracentesis and targeted imaging, followed by prompt surgical intervention, is essential to prevent misdiagnosis, avoid unnecessary dialysis, and ensure full recovery. Clinicians should maintain a high index of suspicion for bladder injury in trauma patients with unexplained azotemia and ascites.
